# Screening for the Most Suitable Reference Genes for Gene Expression Studies in Equine Milk Somatic Cells

**DOI:** 10.1371/journal.pone.0139688

**Published:** 2015-10-05

**Authors:** Jakub Cieslak, Mariusz Mackowski, Grazyna Czyzak-Runowska, Jacek Wojtowski, Kamila Puppel, Beata Kuczynska, Piotr Pawlak

**Affiliations:** 1 Department of Horse Breeding, Poznan University of Life Sciences, Poznan, Poland; 2 Department of Small Mammals Breeding and Raw Materials of Animal Origin, Poznan University of Life Sciences, Poznan, Poland; 3 Department of Animal Science, Cattle Breeding Division, Warsaw University of Life Sciences, Warsaw, Poland; 4 Department of Genetics and Animal Breeding, Poznan University of Life Sciences, Poznan, Poland; University of Lleida, SPAIN

## Abstract

Apart from the well-known role of somatic cell count as a parameter reflecting the inflammatory status of the mammary gland, the composition of cells isolated from milk is considered as a valuable material for gene expression studies in mammals. Due to its unique composition, in recent years an increasing interest in mare's milk consumption has been observed. Thus, investigating the genetic background of horse’s milk variability presents and interesting study model. Relying on 39 milk samples collected from mares representing three breeds (Polish Primitive Horse, Polish Cold-blooded Horse, Polish Warmblood Horse) we aimed to investigate the utility of equine milk somatic cells as a source of mRNA and to screen the best reference genes for RT-qPCR using geNorm and NormFinder algorithms. The results showed that despite relatively low somatic cell counts in mare's milk, the amount and the quality of the extracted RNA are sufficient for gene expression studies. The analysis of the utility of 7 potential reference genes for RT-qPCR experiments for the normalization of equine milk somatic cells revealed some differences between the outcomes of the applied algorithms, although in both cases the *KRT8* and *TOP2B* genes were pointed as the most stable. Analysis by geNorm showed that the combination of 4 reference genes (*ACTB*, *GAPDH*, *TOP2B* and *KRT8*) is required for apropriate RT-qPCR experiments normalization, whereas NormFinder algorithm pointed the combination of *KRT8* and *RPS9* genes as the most suitable. The trial study of the relative transcript abundance of the beta-casein gene with the use of various types and numbers of internal control genes confirmed once again that the selection of proper reference gene combinations is crucial for the final results of each real-time PCR experiment.

## Introduction

For many years milk somatic cell count (SCC) has been used as a major predictor of milk quality, due to its association with the inflammatory status of the mammary gland. Moreover, numerous independent experiments revealed that milk somatic cells (MSC) may be considered as a source of valuable material for gene expression studies, which may reflects the transcriptional potential of the lactating mammary gland [1–3]. It is particularly important for the investigations focused on searching for molecular markers associated with milk composition, as well as studies regarding molecular aspects of immunological response during mammary gland inflammation [4,5]. As it was indicated by Cánovas et al. (2014) [2] in comparison to the other sources (e.g. mammary gland tissue, laser dissected mammary epithelial cells), the quality of the total RNA extracted from the milk somatic cells was high. Moreover, the expression profile of genes investigated in MSC derived material was highly correlated (range: r = 0.80 to r = 0.95) with the expression pattern observed directly in laser dissected mammary epithelial cells.

Due to its unique chemical composition, including its high similarity to human breast milk and significant amounts of bioactive substances, mare's milk is considered as a valuable product for human nutrition [6]. Therefore, studies regarding the genetic background of horse milk composition variability are needed. There have been a few reports describing the nucleotide sequences and the interbreed distribution of genetic polymorphisms of selected equine milk protein genes, e.g. Selvaggi et al. (2010) [7], but there are no available studies focused on their relationship between the milk composition traits and the expression levels of particular genes.

Quantitative real-time PCR (RT-qPCR) technique is a useful tool mainly applied to evaluate the relative transcription levels of genes. Due to its difficult optimization and high sensitivity results obtained from real-time PCR studies are often significantly influenced [8]. Based on previous investigations regarding utility of milk somatic cells derived from different ruminant species (cattle, the goat, sheep, buffalo and the yak) in gene expression experiments [3,4,9–12], we performed this study on equine milk somatic cells and compared the obtained results with the above mentioned studies regarding other species. Additionally, another aim was to select the most appropriate reference genes for normalization of further RT-qPCR experiments on equine MSC.

## Material and Methods

Altogether 39 milk samples were collected from mares representing three different breeds (Polish Primitive Horse, also known as Polish Konik n = 13, Polish Warmblood Horse n = 13 and Polish Cold-blooded Horse n = 13) at 5^th^ week after foaling. Animals (multiparous mares, in 2^nd^-6^th^ lactation) from 3 Polish national horse studs (Kobylniki, Nowe Jankowice and Racot) were kept under similar environmental conditions. Mares were milked manually in the morning (7–9 am). During milking, mothers and their foals remained in visual contact. The obtained milk samples (ca. 100 ml each) were collected to several 15 ml tubes and immediately frozen—in liquid nitrogen (for gene expression studies) and some in -20°C (for biochemical analyses—e.g. beta-casein protein concentration). At the time of sample collection the horses were under veterinary control and did not manifest any disease symptoms. The study was approved by the National Commission for the Ethics of Animal Experimentation, Local Ethics Committee for Animal Research (Poznan, Poland), permission number: 39/2012.

For RNA isolation 5 ml of milk samples frozen in liquid nitrogen were used. After slow thawing the milk was centrifuged in 15 ml tubes for 10 minutes at 4000 x *g*. The supernatant was discarded, while the pellet was dissolved in 1ml TriPure reagent (Roche). The following steps of RNA isolation were performed according to the manufacturer’s protocol. Briefly, after 5 min incubation of the pellet in TriPure 200 μl of chloroform were added and shaken vigorously for 30 s. After 10min incubation at room temperature the sample was centrifuged for 15 min at 12,000 x g. The upper, clear phase was transferred to 0.5 ml isopropanol and incubated for 8 minutes at RT. The next steps involved purification of RNA by 75% ethanol wash and drying of RNA on a 40°C thermoblock. After resuspension of RNA in DEPC treated water, the concentration and A260/280 ratio were measured with a NanoDrop spectrophotometer (Thermo Scientific, USA). RNA integrity was validated by agarose gel electrophoresis (S1 Fig). Reverse transcription reaction (RT) was done using an iScript cDNA Synthesis Kit (Bio-Rad, USA) according to the manufacturer's protocol. Each sample contained an equal concentration of RNA (500 ng). Briefly, iScript reaction buffer (4μl) and reverse transcriptase (1μl), water and RNA were mixed in a final volume of 20 μl. The RT conditions were as follows: 25°C for 5 min, followed by 42°C for 45 min and 85°C for 5 min. Obtained cDNA was stored at -20°C until further analyses. To confirm that our study was not affected by gDNA contamination we performed an additional minus RT negative control analysis (including 5 randomly selected samples derived from each horse breed). The minus RT analysis was done for 3 genes: *RN18S* (single-exon gene), *GAPDH* (the shortest intron separating designed primers) and *ACTB*, using conditions described above for reverse transcription, but without but without the addition of reverse transcriptase. After amplification, the minus RT samples (and the identical number of positive controls) were separated in 1.5% agarose gel (120V, 40 min.) stained with ethidium bromide (S2 Fig).

Primer pairs for RT-qPCR amplification were designed using the Primer3 online tool [13], and synthesized by Sigma-Aldrich (USA). With the exception of *RN18S* (which is the single-exon gene), primers were designed to span exon-exon junctions or were placed in different exons (separated by possibly long intron) to make putative amplification of the genomic DNA impossible (or easy detectable by melting curves analysis). Standard curves were generated as serial 10-fold dilutions of each amplicon (range: 10^8^–10^2^ copies). RT-qPCR amplification was run in duplicates in a LightCycler 480 instrument (Roche Diagnostics, Germany) using the SsoFast EvaGreen Supermix (Bio-Rad, USA) enabling both PCR and signal detection. The RT-qPCR mix (10 μl per sample) contained: 2 μl of nuclease free water, 2 μl of primers mix (0,5 μM each), 5 μl of SsoFast EvaGreen Supermix and 1 μl of cDNA. The detailed RT-qPCR conditions were as follows: 95°C, 2 min. (pre-incubation); 45 cycles of: 95°C, 5 s. (denaturation); 60°C, 12 s. (primers annealing and elongation); 65–97°C (PCR products melting). For each RT-qPCR run, the negative control sample (without cDNA) was included. After each analysis, melting curves were checked to exclude the potential samples contamination (S3 Fig). Primer sequences and cycling details are shown in Table 1.

**Table 1:** Primer sequences and cycling details for RT-qPCR analyses.

Gene name	Primer sequence	PCR product size (bp)	Anneal. temp. (°C)	Mean Cp	Mean SD of the replicates	Ampl. effic.	Slope	y intercept	Source sequence (GenBank)	Genomic location of primers*
*ACTB*	F: tccttcctgggcatggaatc	145	60	23.06	0.352	1.973	-3.389	34.73	NM_001081838	793–812
	R: tcctgtcggcgatgcct									921–937
*KRT8*	F: acccaggagaaggagcaga	108	60	28.83	0.248	1.977	-3.378	39.16	XM_005614767	361–379
	R: gctccacttggtctccagaa									449–468
*GAPDH*	F: gaggaccaggttgtctcctgc	101	60	24.97	0.338	2.002	-3.317	36.69	NM_001163856	891–911
	R: atgagcttgacaaagtggtcgtt									969–991
*RN18S*	F: tttcgatggtagtcgctgtg	101	60	9.93	0.199	1.907	-3.568	40.9	NW_001876670	345–364
	R: cttggatgtggtagccgttt									426–445
*RPS9*	F: gtgaggtctggagggtcaa	160	60	26.40	0.396	2.002	-3.318	37.9	XM_001488024	228–246
	R: agcttcatcttgccctcgt									369–387
*TOP2B*	F: tgcagctgacaataaacag	101	60	31.54	0.428	1.883	-3.349	39.84	XM_005601053	276–294
	R: tgcctttcccattattccaa									357–376
*YWHAZ*	F: tgaagccattgctgaacttg	128	60	28.22	0.234	1.989	-3.980	42.59	XM_001492988	870–889
	R: ctgcttcagcttcgtctcct									978–997
*CSN2*	F: cagcaaagagaggttgaacgc	114	60	10.82	0.231	1.935	-3.487	40.90	NM_001081852	215–235
	R: caggatgctttgtggaacgac									308–328

* all primers are located in the CDS of investigated genes.

Additionally, milk beta-casein concentration was measured using Agilent 1100 Series reverse phase high-performance liquid chromatograph (Agilent Technologies, Germany), according to the methodology described previously by Puppel et al. (2014) [14]

Taking into consideration that there are many significant differences in the lactation parameters (e.g. lactation curve shape, lactation duration, milk yield and composition, somatic cells count) between ruminant and non-ruminant species, we decided to not directly extrapolate results of previously published investigations (regarding selection of the best reference genes) to our study. Thus, based on our experience and literature data [5,9,15] we have selected 8 potential reference genes for normalization of RT-qPCR experiments on mare's milk somatic cells: *ACTB*—actin, beta; *GAPDH*—glyceraldehyde-3-phosphate dehydrogenase; TBP—*TATA* box binding protein; *YWHAZ*—tyrosine 3-monooxygenase/tryptophan 5-monooxygenase activation protein, zeta; *TOP2B*—topoisomerase (DNA) II beta; *RN18S*—18S ribosomal RNA; *RPS9*—ribosomal protein S9 and *KRT8*—keratin 8. Stability of the candidate reference genes and their usefulness in normalization of the equine MSC RT-qPCR experiments were examined (after logarithmic transformation of data) by the geNorm (a part of qbase^PLUS^ software) and the NormFinder bioinformatic tools, which use different approaches of the reference gene selection [16,17]. It should be stressed that our study was designed specifically to meet the requirements of both algorithms, such as the sufficient number of animals, the number of RT-qPCR replicates and the proper number of tested reference genes. We also decided to check whether the selection of the different type and number of reference genes can affect results and conclusions. Thus, we have assessed the interbreed differences in the equine beta-casein gene *(CSN2)* relative transcript abundance with the application of the single reference gene and the geometric mean of two or more reference genes identified by both bioinformatic tools, as the most suitable to normalize our RT-qPCR experiment. Afterwards, we have compared the obtained results by statistical analysis conducted in the IBM SPSS Statistics for Windows package (version 22.0). Between-breed variation of the *CSN2* gene relative transcription level was assessed using non-parametric Kruskal-Wallis rank sum test. If a significant effect of the horse breed was observed, a pairwise comparison (Dunn test) was applied to estimate the exact interbreed differences. Between-breed differences in milk beta-casein concentration were estimated using ANOVA and Tukey’s post-hoc test. Relationship between the CSN2 relative transcript abundance and milk beta-casein amount was examined using Pearson correlation coefficient.

## Results and Discussion

The average number of milk somatic cells ranged between 15 x 10^3^ (± 3.6 x 10^3^) / mL for the Polish Warmblood Horse to 18 x 10^3^ (± 6.6 x 10^3^) / mL for the Polish Cold-blooded Horse breed, although observed interbreed differences were not statistically significant. Our results correspond to those presented by Čagalj et al. (2014) [18] for the Croatian Coldblood Horse breed (19 x 10^3^ / mL^)^ and are within the range of SCC values reported by other authors [19,20,21,22].

The concentration of RNA isolated from 5 ml milk samples varied between different individuals, but generally it was sufficient for reverse transcription and RT-qPCR analyses (mean concentration: 377.6 ± 55.3 ng/μl per each 15μl sample). The A260/A280 ratio for all investigated samples ranged between 1.7 and 1.94.

Taking into account the fact that, to our knowledge, the present investigation is the first one, to asses relative gene expression levels in equine milk somatic cells, we decided to perform a detailed selection of potential reference genes using two bioinformatic algorithms: geNorm and NormFinder. At the beginning of our experiment we observed that usefulness of the *TBP* gene in RT-qPCR normalization is limited due to its low expression in MSC (Cp > 36), and thus we decided to exclude this gene from our study. It should also be explained that *KRT8* is not a classical reference gene, but it is a useful marker of epithelial cells number. Mammary epithelial cells are the most important cells population within the context of major milk proteins expression [23,24].

**Fig 1:**
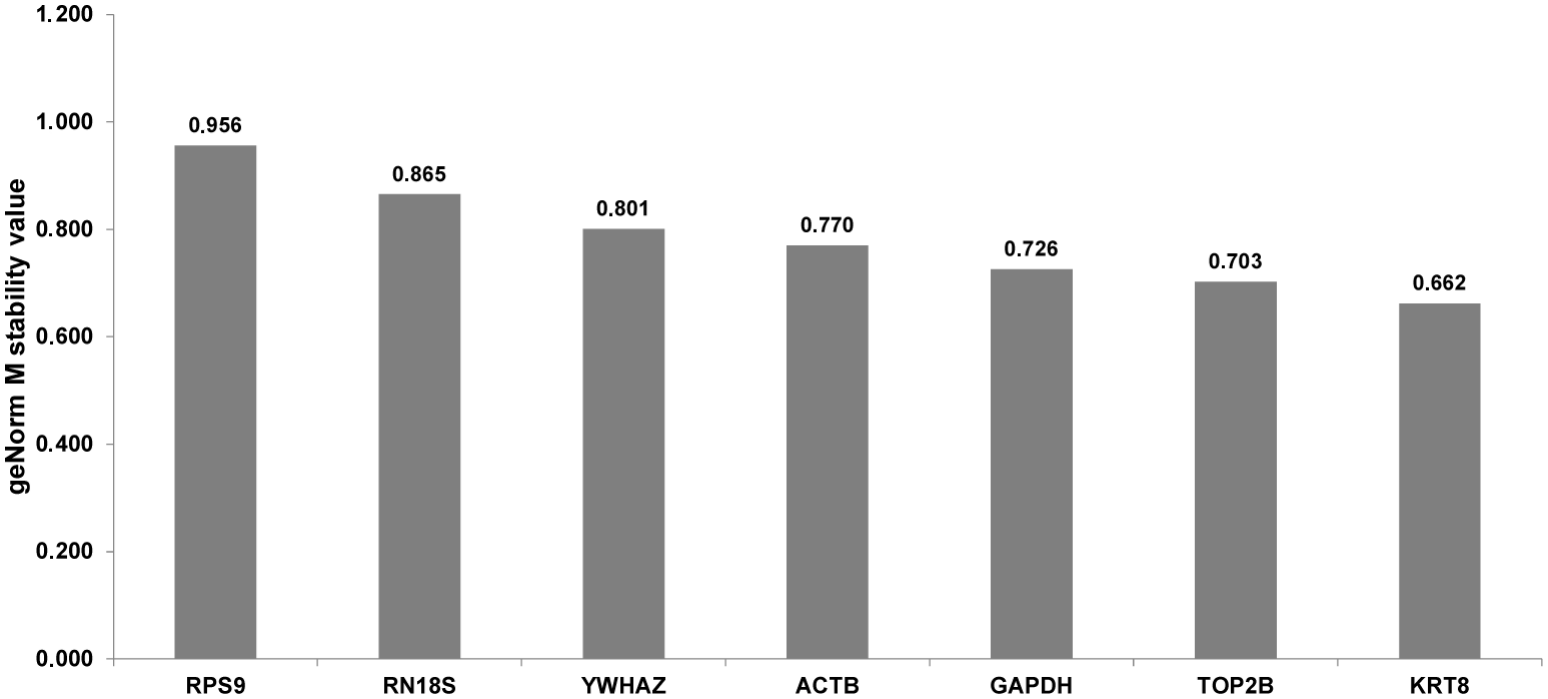
Results obtained by the geNorm algorithm. M stability value for all genes studied (more stable genes represent lower M values).

**Fig 2:**
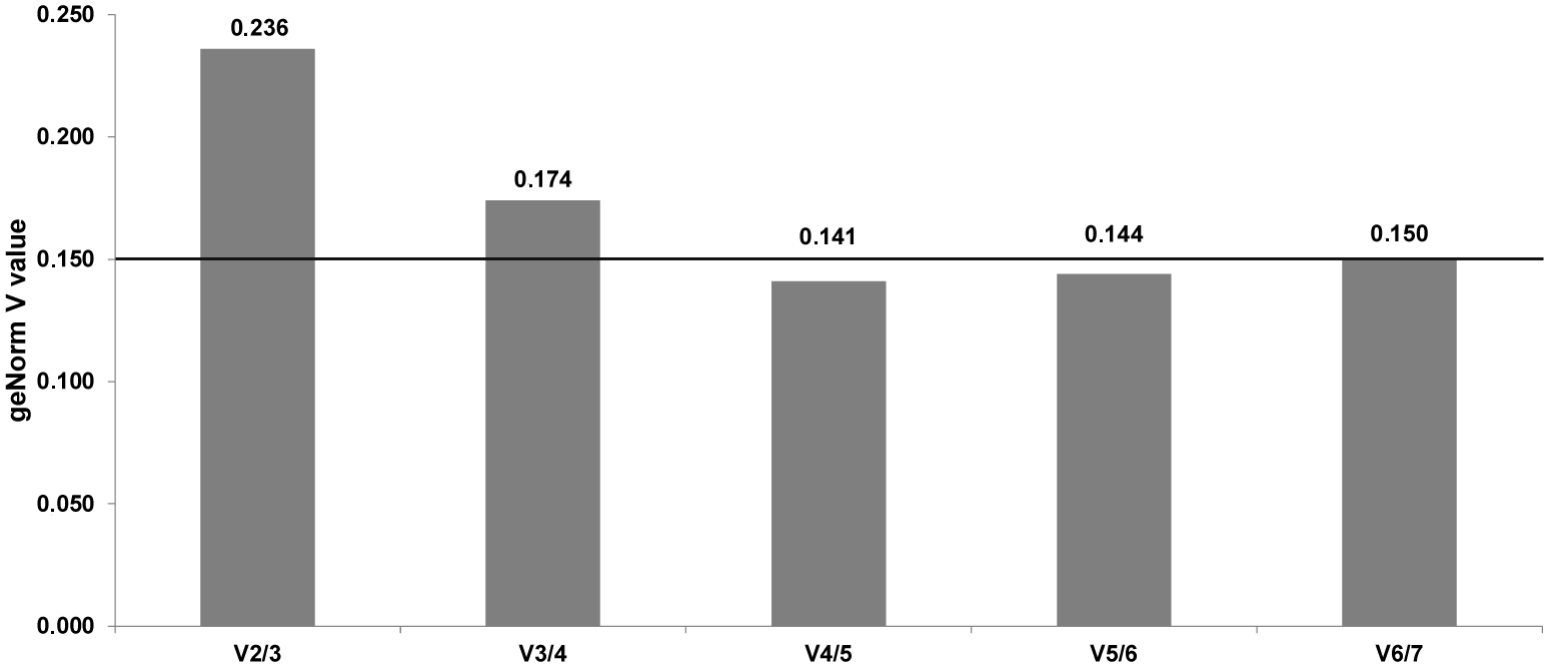
Results obtained by the geNorm algorithm. Pairwise variation of relative transcription levels for studied genes (combinations which do not exceed the treshold of V = 0.150 are useful in present RT-qPCR study normalization).

**Fig 3:**
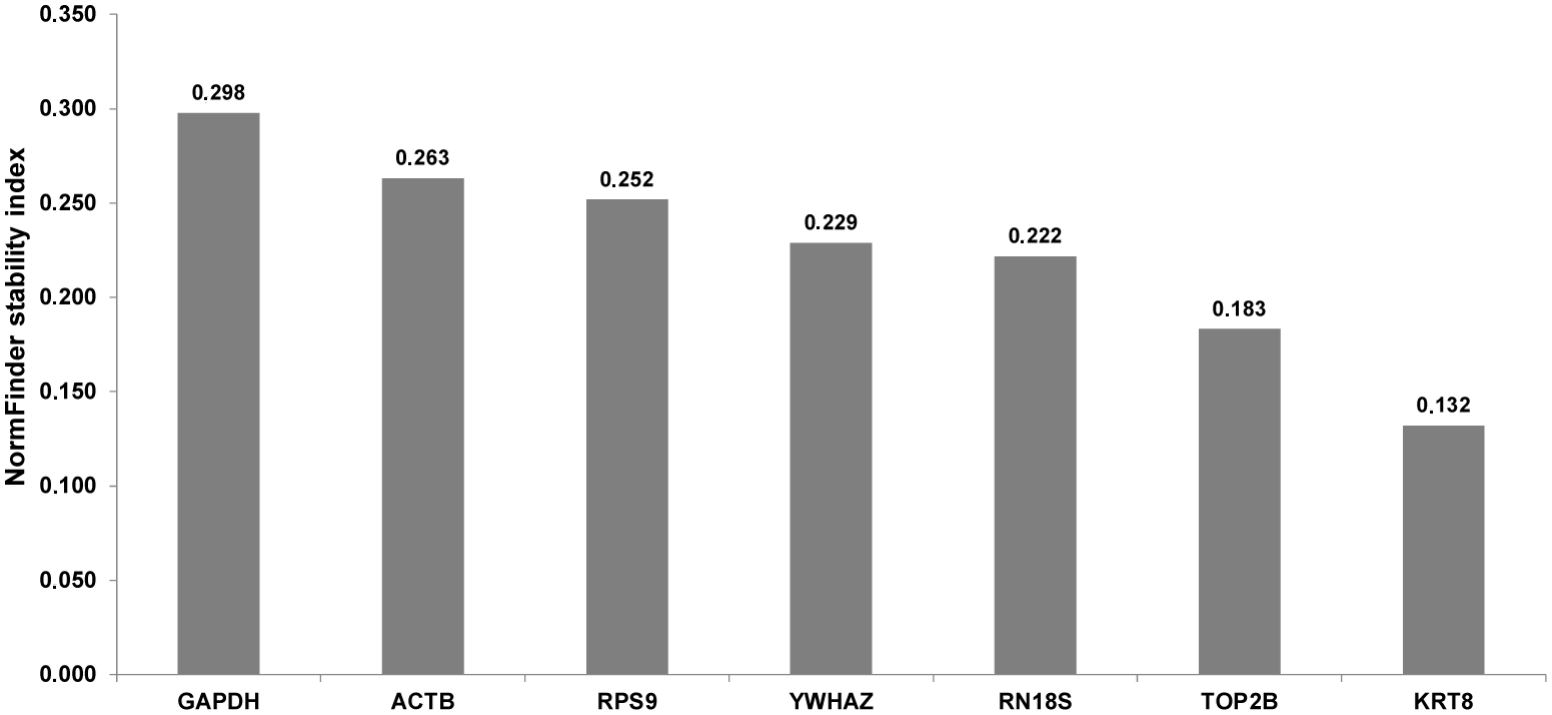
Results obtained by the NormFinder algorithm. The lower stability index is calculated for genes presenting greater stability.

Because of various statistical approaches used in different tools when looking for the most stable reference genes it is likely, that the final results may differ [8]. In the case of our study, analysis by geNorm showed moderate stability of all investigated candidate genes, with the mean M value = 0.783 (Fig 1). Generally, none of the tested candidate reference genes exceed the M value = 1, which means that their stability is acceptable for the normalization of RT-qPCR analyses on MSC [25]. Analysis by the geNorm algorithm pointed out that *KRT8* and *TOP2B* are the most stable genes (M value = 0.662 and 0.703, respectively), whereas the lowest stability (M = 0.956) was recorded for the *RPS9* gene (Fig 1). Pairwise comparison of normalization factors for candidate reference genes showed that in the case of three combinations *(ACTB* + *GAPDH*; *GAPDH* + *TOP2B* and *TOP2B* + *KRT8*) variation (V value) did not exceed 0.150 (Fig 2) and according to geNorm the geometric mean of expression for the above mentioned 4 reference genes is required for proper normalization of our RT-qPCR experiment. Similarly to geNorm, analysis by the NormFinder algorithm (with the use of the horse breed as grouping factor) revealed the best stability index for the *KRT8* and *TOP2B* genes (0.132 and 0.183, respectively). At the opposite end of the stability ranking (stability index = 0.298), the *GAPDH* gene was the least stable (Fig 3). Interestingly, instead of two most stable candidates *(KRT8* and *TOP2B*) as the most valuable combination of reference genes for normalization of our RT-qPCR study *TOP2B* and *RPS9* were pointed out by NormFinder. In the case of this algorithm the inter- and intragroup variation in candidate reference gene expression is calculated to find the most stable ones [17]. Sometimes (e.g. in the present study) the optimal pair does not include the most stable gene. It may be related to the necessity to compensate for the overexpression of the one reference gene by another one, which is underexpressed in the same group [26]. Detailed results regarding the intra- and intergroup variation of investigated reference genes calculated by NormFinder are shown as supplementary data (S4 Fig).

A detailed comparison of results recorded by us and by other authors, revealed species specific differences. For example, the *RPS9* gene, which in this study was pointed by geNorm as the least stable (but good in a combination with the *TOP2B* gene according to the NormFinder algorithm), is generally suggested to be a very useful internal control gene for the normalization of RT-qPCR experiments on milk somatic cells and on lactating mammary glands of several ruminant species, e.g. cattle, the buffalo and the yak [9,12,27]. However, it should be noticed that there are some studies showing that this gene can be potentially co-regulated with commonly applied normalization genes: *ACTB* and *GAPDH* [27], and thus, should be used in combinations very carefully. In our study these two genes were placed almost at the opposite ends of the stability rankings generated by two algorithms: geNorm and NormFinder (Figs 1 and 3). In the case of geNorm, the *ACTB* and *GAPDH* genes were indicated as stable and necessary for proper normalization of our RT-qPCR experiment, in combination with two other genes (*KRT8* and *TOP2B)*. These results are in agreement with previously published studies on zebu and buffalo milk somatic cells [11,12]. On the other hand, studies on yak MSC and on material received from mammary gland biopsies in cattle showed that the expression of *ACTB* and *GAPDH* is unstable and these two genes are not recommended for normalization of RT-qPCR experiments [9,27]. We should also stress that in our experiment both bioinformatic algorithms calculated the highest stability value for the *KRT8* gene. Thus, it seems to be justified to use it for RT-qPCR normalization as a valuable marker of epithelial cell number, as it was suggested by other researchers [5,24]. Interestingly, we did not find the *TOP2B* gene, which in our experiment was pointed out by both algorithms as very stable, in any other investigations regarding RT-qPCR normalization on milk somatic cells. Because its relatively low expression level (mean Cp = 31.54) the utility of *TOP2B* gene in RT-qPCR normalization can be undermined, since the well known sensitivity of such genes to the technical variation, e.g. pipetting errors and/or unstable amplification efficiency [28]. Nevertheless, using this gene in a combination with other reference genes should stay under consideration. Our pilot study (data not shown) has indicated that instead of high-copy transcripts observed in the equine milk somatic cells (e.g. encoding caseins), we can also expect the low abundant mRNAs encoding important milk proteins, like lysozyme or lactoferrin (Cp~30). Therefore, it should be scaled, whether using the *TOP2B* gene in the 3–4 reference genes set, does not make it much “universal” for gene expression experiments on MSC.

**Fig 4:**
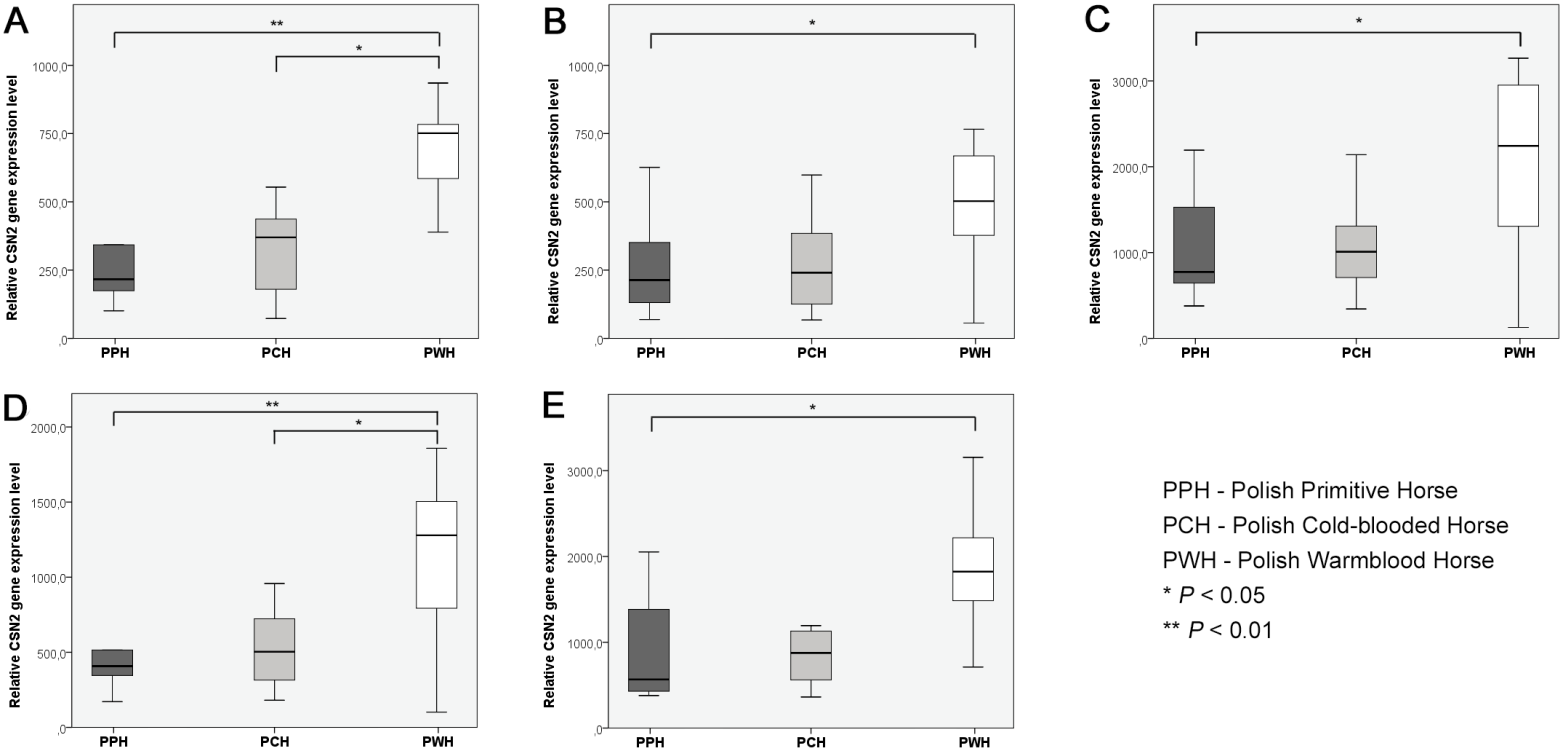
The effect of selection of different types and numbers of reference genes on beta-casein *(CSN2)* gene relative transcription level in equine milk somatic cells. Reference gene combinations: A − *KRT8 + TOP2B + ACTB + GAPDH* (the best combination according to geNORM); B − *KRT8 + TOP2B +ACTB*; C − *KRT8 + TOP2B*, D − *TOP2B + RPS9* (the best combination according to NormFinder), E − *KRT8* (alone—the most stable reference gene according to both algorithms).

Unfortunately, we have to agree with many previously published papers, in which the simple conclusion was included: “The ideal reference gene for RT-qPCR normalization does not exist” [16,29]. Moreover, ambiguities observed in the results recorded in our and other authors’ studies forced us to consider once again, which model of screening for the most suitable reference genes is correct and where the potential discrepancies come from. Therefore, each particular experiment should be preceded by detailed validation of the candidate reference genes set. However, there is also no simple answer to the question which algorithm selecting the most suitable reference genes should be applied. In our study we used the geNorm and NormFinder algorithms, which fundamentals were described in detail by Vandesompele et al. (2002) [16] and Andersen et al. (2004) [17]. As it was mentioned above, recorded results were not fully compatible and thus, we performed a pilot experiment to verify whether in our particular study the selection of proper types and numbers of normalization genes is really important. To confirm this we assessed interbreed differences in the beta-casein *(CSN2)* gene relative transcription level in milk somatic cells of three horse breeds (Polish Primitive Horse, Polish Cold-blooded Horse and Polish Warmblood Horse), using five different combinations of reference genes: *KRT8 + TOP2B + GAPDH + ACTB* (the best combination according to geNorm), *KRT8 + TOP2B + GAPDH*, *KRT8 + TOP2B*, *TOP2B + RPS9* (the best combination according to NormFinder) and *KRT8* (alone). Although the observed tendencies were similar for all the scenarios (with the highest relative *CSN2* transcript abundance in the Polish Warmblood Horse), we noticed an interesting variability in statistical significance levels (Fig 4). The difference in the *CSN2* relative transcription level between the PPH and PWH breeds was observed in all configurations, although in the case of scenarios regarding the best reference gene combinations according to geNorm and NormFinder (Fig 4A and 4D), the statistical significance was evident (*P* < 0.01 *vs*. *P* < 0.05 in all other comparisons). Additionally, in the case of the above mentioned comparisons (best combinations obtained with the application of both algorithms) the statistically significant (*P* < 0.05) difference in the *CSN2* relative transcript abundance was observed also between the PCH and PWH breeds. The difference was not significant in the other adopted scenarios. This suggest that in our experiment, despite different results obtained by two algorithms (regarding the type and number of reference genes) their application improves sensitivity of the method. We should also underline that the randomly selected gene of interest *(CSN2)* revealed to be about 3000 fold highly expressed than majority of investigated reference genes, which may potentially alter obtained results, since it is recommended to use internal control genes of similar expression to genes of interest. On the other hand, results obtained in our project (unpublished data) show, that in the equine MSC we can expect numerous transcripts of moderate or even low expression level for which the above described reference genes set would be more appropriate.

Analysis of the milk beta-casein concentration showed the highest protein content (1.74±0.20 g/L) in the case of Polish Primitive Horse breed, whereas the lowest CSN2 level (1.22 ±0.07 g/L) was recorded for Polish Cold-blooded Horses (*P*<0.05) (S5 Fig). No significant correlation was noticed between CSN2 transcript level measured in MSC and milk beta-casein concentration.

## Conclusions

Our investigation has indicated that equine milk somatic cells are valuable sources of mRNA for RT-qPCR analyses, but require an individual normalization, since the results obtained for other species do not correlate entirely. We also confirmed once again that proper selection and evaluation of potential reference genes is one of the most important steps and should be conducted before each new real-time PCR experiment.

## Supporting Information

S1 Fig RNA integrity control. (TIF)

S2 Fig The minus RT control for *ACTB*, *GAPDH* and *RN18S* genes. (TIF)

S3 Fig Melting curves generated for each amplified gene. (TIF)

S4 Fig Inter- and intra-group variation of investigated reference genes, according to NormFinder algorithm (columns represent the intergroup variation and the error bars represent the intra-group variation). (TIF)

S5 Fig Between-breed comparison of the milk beta-casein (CSN2) concentration (*P<0.05). (TIF)
