# Supplementary figures and images for: Screening for the Most Suitable Reference Genes for Gene Expression Studies in Equine Milk Somatic Cells
Source: PLoS One. 2015 Oct 5;10(10):e0139688. doi: 10.1371/journal.pone.0139688 (PMC4593561; doi:10.1371/journal.pone.0139688)

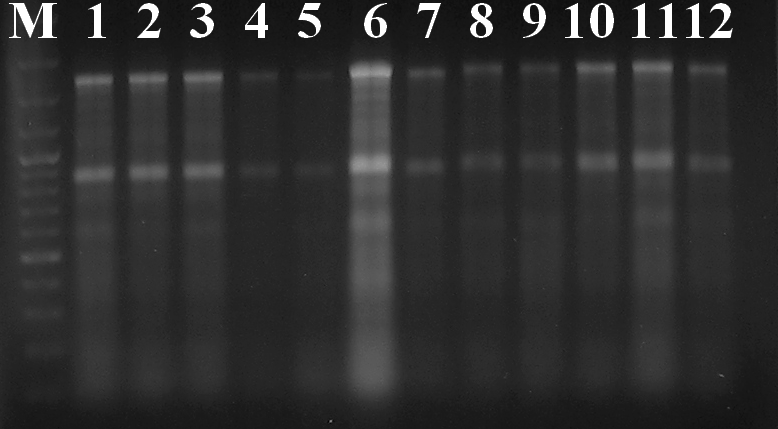

Supplement: S1 Fig — (TIF) [file pone.0139688.s001.tif]

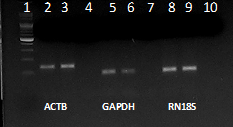

Supplement: S2 Fig — (TIF) [file pone.0139688.s002.tif]

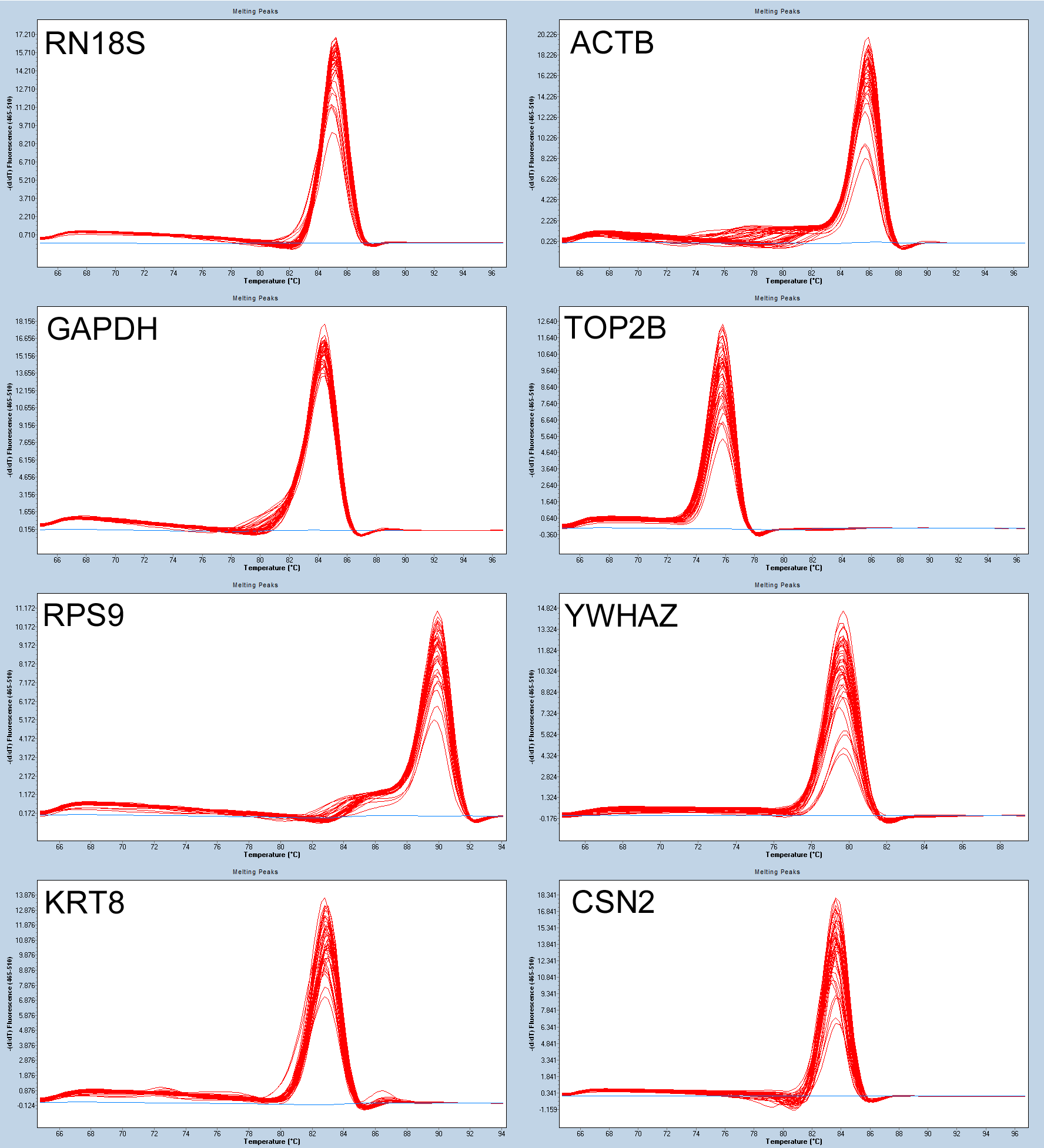

Supplement: S3 Fig — (TIF) [file pone.0139688.s003.tif]

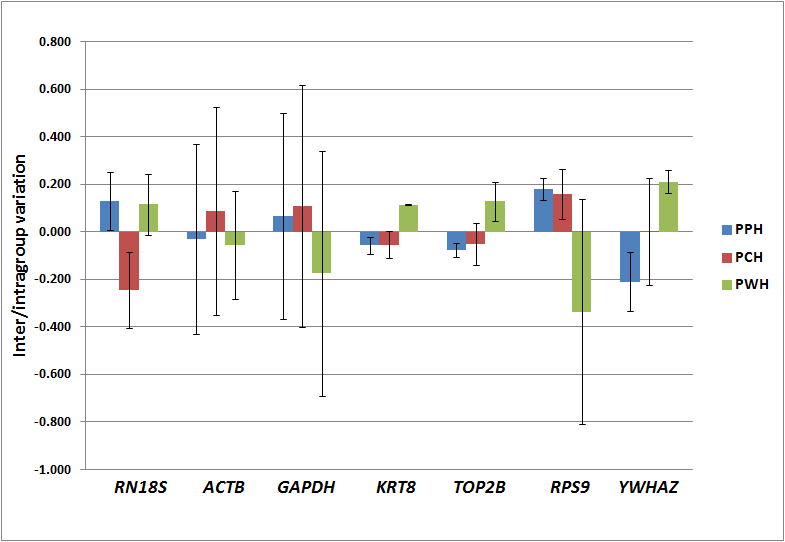

Supplement: S4 Fig — (TIF) [file pone.0139688.s004.tif]

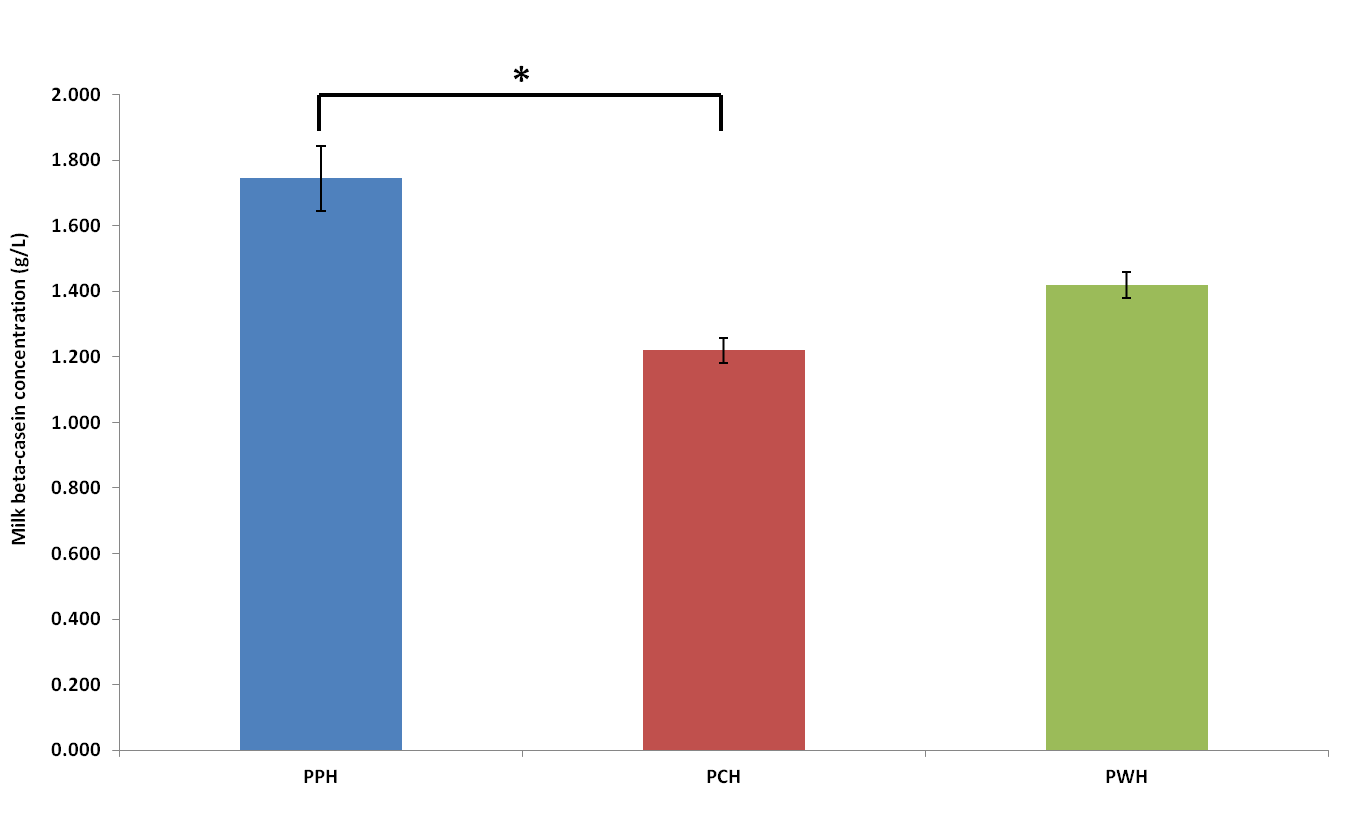

Supplement: S5 Fig — (TIF) [file pone.0139688.s005.tif]
